# Re: “Re: Diagnostic Accuracy of Global Pharma Health Fund Minilab^™^ in Assessing Pharmacopoeial Quality of Antimicrobials”

**DOI:** 10.4269/ajtmh.18-0127b

**Published:** 2018-06

**Authors:** Hui Pan, William Ba-Thein

**Affiliations:** Shantou-Oxford Clinical Research Unit; Shantou University Medical College; Shantou, Guangdong, People’s Republic of China; E-mail: michaelhuipan@163.com; Shantou-Oxford Clinical Research Unit; Shantou University Medical College; Shantou, People’s Republic of China; Department of Microbiology and Immunology; Shantou University Medical College; Shantou, Guangdong, People’s Republic of China; E-mail: wbathein@stu.edu.cn

Dear Sir,

Comments made by Dr. Richard Jähnke^1^ on our recent article^2^ highlighted the role of the Global Pharma Health Fund (GPHF) Minilab^™^ in detecting falsified medicines, without addressing poor-quality medicines and their associated safety concerns, as presented in our article.^2^

We did not “imply that the Minilab was intended to replace high performance liquid chromatography (HPLC) for the assessment of drugs.” Indeed, designed as a semi-quantitative test kit, the Minilab cannot replace HPLC, which is the gold standard quantitative method accepted and used by pharmacopeias worldwide. As clearly mentioned in the article,^2^ our objective was to evaluate the Minilab’s diagnostic accuracy in monitoring the drug quality of antimicrobials using HPLC as the reference standard because related information was not available in the literature, Minilab manuals, or the GPHF website.^3^ Testing against a well-recognized reference test is the standard procedure to evaluate the characteristics of any diagnostic technique; the aim was not to “compare high-tech and low-tech options” as suggested by Dr. Jähnke.

By having only the lower active pharmaceutical ingredient (API) limit (80% of the reference standard) but not any upper API limit as “out of range,” the Minilab system will miss poor-quality drugs, especially those with APIs higher than the standard. Therefore, understanding the diagnostic accuracy of the Minilab system is important for making informed decisions to use the Minilab either as a stand-alone or non–stand-alone screening system for falsified and/or poor-quality drugs. However, without knowing its diagnostic accuracy, more than 800 Minilab kits have been deployed in 97 countries worldwide,^3^ and these have been used inappropriately in some countries. For example, the Minilab was used improperly as the only test for the surveillance of drug quality in India.^4^ Thus, studies investigating its diagnostic accuracy, like ours,^2^ are urgently needed. With the observed poor diagnostic accuracy of the Minilab,^2^ there is a possibility of underestimating the prevalence of poor-quality antimicrobials by using the Minilab system as the only screening test or when only a portion of Minilab-tested samples are confirmed by HPLC.

The study cited to support the contention that “a combination of both HPLC and Minilab could reduce costs and maintain comprehensive, quality surveillance,”^5^ did not use HPLC at all, and their definition of substandard medicines was inaccurate.^5^ That is, a sample that failed any of the inspections/tests was regarded as substandard, regardless of further confirmatory testing. A combination of the Minilab and HPLC, in fact, could be helpful for monitoring falsified medicines and reducing costs, but this approach might not prove reliable for detecting poor-quality medicines, and, thus, might waste resources and compromise patient safety, especially in developing countries.
